# Treatment of Dysentery by Enemata of Corrosive Sublimate, Etc.

**Published:** 1890-07

**Authors:** 


### TREATMENT OF DYSENTERY BY ENEMATA OF CORROSIVE SUBLIMATE, ETC.

It is now generally recognized that certain morbid conditions of the intestinal tract may be favorably modified by various drugs belonging to the class of antiseptics, among which the chief are calomel, bismuth, naphthalin and thymol. It is a noteworthy fact that these substances are insoluble, and it is in virtue of this property that they are enabled to run the gauntlet of the absorbents and exert their specific action upon the intestinal contents. The best of all antiseptics, corrosive sublimate, has thus far been of little use for the purpose mentioned, because it was supposed that no benefit could be exerted by any but a lethal dose. While this may be true of its administration *per os*, it is shown by G. Lemoine (*Bulletin general de Therapeutique*, January, 1890) to be a mistake so far as concerns administration *per rectum*.

Lemoine has treated 54 cases of dysentery by enemata of corrosive sublimate, and with the happiest results. The strength of the solution was one to five thousand, of which two hundred grammes were at first administered three times a day; later, two hundred grammes of a solution of one to three thousand were injected twice daily. Improvement showed itself, as a rule, after the first injection, the first symptoms to disappear being the tormina and tenesmus. In a certain number of cases the tenesmus was so great that the enema could not be administered without a preliminary treatment, which consisted in painting the sphincter with a five per cent. solution of cocaine.

In acute cases, a cure resulted from this treatment in from three to four days; whereas, in the more chronic cases which presented themselves for treatment on account of an acute exacerbation, a cure was effected, as a rule, in one day. The latter statement is somewhat startling in view of the well-known fact that chronic dysentery is decidedly rebellious to all the usual modes of treatment.—*Medical News.*

